# Non-communicable disease multi-morbidity in policies from India, Thailand, and South Africa: A comparative document review

**DOI:** 10.1177/26335565251330371

**Published:** 2025-04-13

**Authors:** Linju Joseph, Rakhal Gaitonde, Charutha Retnakumar, Athira Krishnan, Thoniparambil Ravindranathanpillai Lekha, Neethu Sasidharan, André van Rensburg, Naomi Levitt, Nilawan Upakdee, Jissa Vinoda Thulaseedharan, Mathew Joseph Valamparampil, Sivadasanpillai Harikrishnan, Sheila Greenfield, Paramjit Gill, Justine Davies, Semira Manaseki-Holland, Panniyammakal Jeemon

**Affiliations:** 1 29354 Sree Chitra Tirunal Institute for Medical Sciences and Technology, Achutha Menon Centre for Health Sciences Studies, Trivandrum, India; 2 1724 Institute of Applied Health Research, College of Medical and Dental Sciences, University of Birmingham, Birmingham, UK; 3 Centre for Rural Health, University of KwaZulu-Natal, Durban, South Africa; 4 Division of Endocrinology and Diabetes, University of Cape Town, Cape Town, South Africa; 5 Faculty of Pharmaceutical Sciences, 59212 Naresuan University, Phitsanulok, Thailand; 6 2707 Academic Unit of Primary Care, Warwick Medical School, University of Warwick, Warwick, UK

**Keywords:** multi-morbidity, policy, strategy, non-communicable disease programmes, documents

## Abstract

**Background:**

Over the years, non-communicable diseases (NCDs), as well as the number of people with multiple chronic NCDs or multi-morbidity, are on a sharp rise globally, especially in low and middle-income countries (LMICs). This review attempts to deepen the knowledge (policy landscape) of how managing multiple NCDs and associated challenges are addressed across the health systems policies from India, South Africa and Thailand.

**Methods:**

We conducted a search of two search engines (PubMed and Google) and the websites of national departments from February 2022 to December 2022. An analytical framework was produced for the qualitative document analysis, focusing on definitions of multi-morbidity, potential policy actions at patient, provider, health system, and macro-level domains, including social determinants of health. We utilised framework analysis of the national-level policies and related documents to explore the co-existent nature of multiple NCDs in India, South Africa, and Thailand.

**Results:**

Of the 54 analysed documents, 11 (20.4%) were national policies/ programmes, 15 (27.8%) were operational or implementation or management guidelines, 12 (22.2%) were training manuals, 16 (29.6%) were action plans/ strategic plans/ frameworks. None of the countries had specific policies dealing with NCD multi-morbidity. Findings from the thematic analysis showed that health promotion activities at patient-level targeted multiple risk factors; however self-management support is for specific NCDs such as diabetes.

**Conclusions:**

Our study highlights the need for dedicated policies that adopt a patient-centred and integrated approach with appropriate consideration of social determinants of health and health inequalities within these policies to manage NCD multi-morbidity holistically and effectively.

## Introduction

Over the past few decades, life expectancy has increased globally with dramatic changes in low- and middle-income countries (LMIC) settings.^1^ For example, the average life expectancy in India increased from 63 in 2002 to 70.2 years in 2022.^2^ In parallel with rising life expectancy, the demand for healthcare utilisation also increased owing to the rise in chronic non-communicable diseases (NCD).^3^ A nationally representative cross-sectional study from India reported that the average number of outpatient visits over the previous year rose from 2.2 to 6.2, while the proportion of individuals reporting a hospital stay in the last three years increased from 9% to 29% among those without any NCDs and those with two or more NCDs, respectively. NCDs include cancers, cardiovascular diseases, chronic respiratory diseases, mental, and behavioural conditions and diabetes.^4^ Furthermore, the average years spent in good health is not increasing in LMICs; even worse, it is declining in some countries.^5^ Despite this epidemiological transition, most healthcare systems in LMICs are disease-oriented and still primarily focused on episodic and acute care that is ill-suited for the needs of chronic NCDs.^6–8^ Within the context of rising prevalence and persistent socio-economic disparities prevailing in LMICs, NCDs tend to co-exist together in an individual rather than being seen in isolation.^9,10^ Consequently, multi-morbidity, commonly defined as the co-occurrence of two or more chronic conditions, has emerged as a growing public health concern in LMICs.^11,12^ While the above definition is widely used, there have been criticisms for the lack of reflection on the complexities of living with multi-morbidity.^13^ Several authors suggested that multi-morbidity should be understood as the presence of two or more chronic conditions (whether chronic diseases, acute diseases, bio psychosocial factors, or somatic risk factors) within an individual, along with consideration of various modifiers that influence its effects, such as social networks, healthcare utilisation, disease burden, and coping strategies. The more inclusive definition seeks to capture the complexities and consequences of living with multi-morbidity, providing a foundation for better understanding and addressing them in public health policies.^14,15^

One of the significant challenges faced by patients with multi-morbidity (those accessing care) is the treatment burden due to managing multiple appointments, complicated medication regime, and several lifestyle changes.^16,17^ Therefore, treatment itself can represent an excessive burden for patients with multi-morbidity, alongside their burden of illness.^18^ The growing literature on multi-morbidity treatment burden from LMICs suggests how treatment burden in multi-morbidity is linked with poor quality of life from LMICs.^19,20^ Further, multi-morbidity increases out-of-pocket expenditure for patients from low-resource health systems.^4,21–23^

Given the evidence on poorer health care access and quality of care, managing multi-morbidity necessitates health system redesign in LMICs. The Sustainable Development Goals (SDG)-3 recommends universal health coverage to ensure access to high-quality care across the life course.^24^ The World Health Organization (WHO) also recommends better policies for tackling social determinants of health and increasing awareness of multi-morbidity among policymakers.^25^ Therefore, we selected three countries based on convenience from three WHO regions; Sub-Saharan region (South Africa), South-Asian region (India) and South-East Asia (Thailand) to assess the current national policies on co-existent nature of chronic NCDs. The health system’s structure and financing differ in South Africa, Thailand, and India. For example, Thailand’s universal health coverage (UHC) efforts ensure free primary care for most of the Thai population.^26^ Both India and South Africa launched national health insurance schemes to provide protection against catastrophic out of pocket payments as a step towards UHC and are currently in different phases of implementation.^27,28^

A clear and well-defined policy sets the foundation for proactive care, and in the context of multi-morbidity, it can lead to care that is more integrated, which necessitates the strengthening of primary care. Policy, in this context, is a crucial acknowledgment of the existing problem and serves as an expression of the intent to address it effectively. However, it is important to note that the mere existence of a policy may not automatically lead to significant changes in practice and outcomes.^29^

Within this context, our study aims to synthesise and identify the gaps in the current policies of NCD multi-morbidity management in primary care settings in India, Thailand, and South Africa. Further, we examined policies to gather any lessons that may be adapted for the effective management of NCD multi-morbidity in LMICs. The following research questions were formulated for our review:

• How have the current policies constructed the problem of NCD multi-morbidity, and what type of solutions are being suggested?
• What are some of the policy actions (if any) based on current policies, that can be built upon for a policy on NCD-multi-morbidity at the primary care level?

## Materials and methods

### Study design

We conducted a qualitative document review and analysis^30^ on the national-level policies and policy-related documents relevant to patients with NCD multi-morbidity. Three countries India, Thailand, and South Africa were conveniently sampled to explore how NCD multi-morbidity has been articulated. Further details on NCD burden for the three countries are provided in Online Supplemental Box S1.

### Search strategy and data selection

For this study, we were interested in government health policy documents to synthesise current management of NCD multi-morbidity. We developed a broad search strategy (LJ and CR) and focused on current policy documents, guidelines, action plans, and management frameworks for NCD multi-morbidity management.^15,31^We searched government health department websites and Google search engine between February 2022 to December 2022 without any restrictions on time period. Furthermore, NCD programme managers and health system experts from Thailand, India, and South Africa were consulted to identify policy or guideline documents related to the management of NCD multi-morbidity. We also included recent inter- and intra-departmental consultation documents from the government considered pertinent to informing forthcoming policies, as suggested by programme managers. (Search strategy is provided in the online supplement; Box S2, Additional country wise strategies in Box S3-S4).

Two researchers (LJ and CR) screened potentially eligible documents independently, and clarification of the eligibility of the inclusion of documents was performed with a wider team (PJ and RG). We excluded single-disease clinical practice guidelines, policies with a focus only on tertiary care, expert opinions and editorials, and policy documents superseded by more recent policy documents unless they provided additional crucial information related to the management of multi-morbidity (Table 1).

**Table 1.** Inclusion and exclusion criteria for documents.

Domain	Inclusion criteria	Exclusion criteria
Chronic conditions	National policies addressing chronic NCDs	Single disease clinical practice guidelines
Strengthening of primary care	National policies detailing strengthening of primary care, reorganising care for managing chronic NCDs	Policies addressing tertiary or secondary care alone
Universal health coverage	National policies detailing universal access to healthcare	Expert opinions, editorials or articles mentioning universal health coverage without a national policy

## Analytical framework

**Figure 1.**
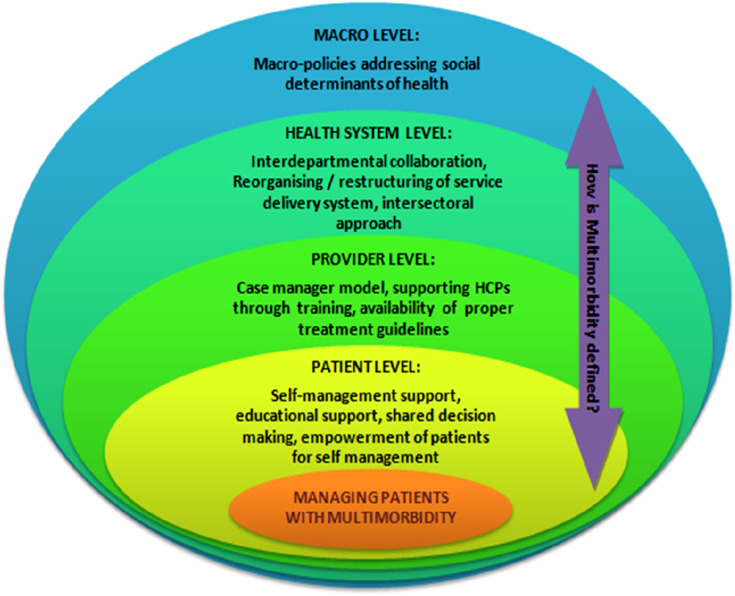
Analytic framework for classifying multi-morbidity management in policies.

We developed an analytical framework (Figure 1) using an iterative approach that involved multiple discussions (LJ, CR, PJ, RG) and a focused literature review of approaches to managing NCD multi-morbidity. The overarching research question was to explore how multi-morbidity has been addressed (if at all) and the problem of multi-morbidity constructed in the policy documents from the three selected countries. Additionally, to identify the gaps in the current policies, we mapped potential strategies either that are suggested in the policy documents directly or that would have potential to manage NCD multi-morbidity. The main categories making up the framework were definitions of NCD multi-morbidity used or implied in the policy document and interventions/strategies (potential) for the management of NCD multi-morbidity categorised as being relevant at the patient, provider, health system and macro levels, including social determinants of health based on the literature.

As defined in the introduction, we explored whether the documents articulated NCD multi-morbidity holistically, i.e., by including the influence of broader social, environmental, aging, and recognition of the complexities of living with multi-morbidity, as they are important in managing multi-morbidity as a public health issue.

At the individual or patient level, we focused on policy directions for self-management support for NCD multi-morbidity.^32^ Furthermore, we explored patient-level strategies that encouraged patient-centred care for self-management, which incorporated personal preferences and functional priorities in the treatment planning of multi-morbidity.^33^

At the provider level, we assessed policy directions that support healthcare providers in delivering integrated care for the management of multi-morbidity. Based on evidence on efficacy from previous studies conducted in high-income countries, we incorporated case manager models that incorporated integrated management of multi-morbidity in our analytical framework.^34,35^ Case manager models incorporate nurses or specific health care workers for preliminary check-ups, including physical and mental health. Further, these models addresses difficulties with current treatment regimens, problems with daily activities and social problems, and support patients to achieve their goals followed by the diagnosis by the physician resulting in an improvement in the quality of life.^34,35^ Interventions for provider support such as training, availability of treatment guidelines, etc., were also included in our analytical framework and explored in the policy review.

For strategies at the organisational or health system levels, we included collaborative teamwork across different departments for the overall management of patients with multi-morbidity. Policy directions that aimed at a whole system change or service reorganisation were incorporated in the analytical framework. For example, a “Multiple Long-term Conditions Care Model” developed by the Joint Action on Chronic Diseases and Promoting Healthy Ageing across the Life Cycle focused on improving health outcomes through quality health care delivery, decision supports, self-management supports, utilisation of information systems and technology, and social and community resources that targeted interventions at the health system level.^36^ Furthermore, we considered the WHO’s framework for 'integrated people-centred health services' that focused on health systems redesign to meet the people’s needs particularly continuity of care in our analytical framework.^26^

For policy actions at the macro level, we considered policies and interventions improving social determinants of health as described by Dahlgren and Whitehead^37^ in our analytical framework. Interventions or policy actions aimed at mitigating upstream factors such as income inequalities, poverty, work-related health hazards, and lack of social cohesion with the aim of preventing or managing NCD multi-morbidity.

## Data analysis

As described, we first developed our analytical framework.^38^ Next, we assessed all retrieved documents to identify the specific policies relevant to the management of multi-morbidity according to the inclusion criteria.^39^ Documents in Thai were translated using Google Translate. Documents from each country were coded according to the categories of the developed analytical framework in an Excel spreadsheet. Two coders (CR/ AK, and LTR/NS) applied the analytical framework. Another coder (LJ) coded randomly selected 10% of the policies for multi-analyst triangulation,^40^ and themes were finalised with discussion with a third analyst (RG). We then examined the results to understand the possible gaps and potential areas for further policy action. We extracted examples from policy documents to illustrate our analysis.

## Results

### Search results

We retrieved 175 potentially eligible documents, of which 54 were relevant to multi-morbidity. Among the 54 documents, 26, 17, and 11 were from India,^41–67^ South Africa,^68–84^ and Thailand,^85–95^ respectively (Online Supplemental Figure S1). The expert teams from South Africa and Thailand provided 14 documents in the review. The included documents had policies/programmes (n=11),^41,45,52,64,68,72,75,81,86,87,95^ operational or implementation or management guidelines (n=15),^42,46,48,54,57–59,69,73,78,84,91,93,94^ training manuals (n=12),^49–51,56,60–63,65,83,85,90^ action plans/frameworks/strategic plans (n=16),^43,44,47,53,55,70,71,74,76–80,82,88,89,92^ at the national level (Table 2). Two state-level NCD programmes from India were also included.^66,67^

**Table 2.** Policy documents from India, Thailand, and South Africa.

Sl. No.	Year	Document title	Responsible organization	Country
1	2010,2013 revised	National programme for prevention and control of cancer, diabetes, cardiovascular diseases and stroke	Ministry of health and family welfare	India
2	2011	Operational guidelines-National programme for health care of the elderly	NRHM, Ministry of health and family welfare	India
3	2012	Twelfth Five-year plan (2012–2017)	Planning Commission, government of India	India
4	2012	National action plan and monitoring framework for prevention and control of NCDs	Ministry of health and family welfare	India
5	2016	Operational framework management of common cancers	Ministry of health and family welfare	India
6	2016	Standard treatment guidelines- hypertension screening, diagnosis, assessment, and management of primary hypertension in adults in India	Ministry of health and family welfare	India
7	2016	Operational guidelines on prevention, screening and control of common NCDs	Ministry of health and family welfare	India
8	2016	Training manual for medical Officers on reducing risk factors of NCDs	Ministry of health and family welfare	India
9	2016	Training manual for community health workers on reducing risk factors of NCDs	Ministry of health and family welfare	India
10	2017	National health policy	Ministry of health and family welfare	India
11	2017	National multi-sectoral action plan for prevention and control of common non-communicable disease	Ministry of health and family welfare	India
12	2017	National framework for Joint TB-diabetes collaborative activities	Ministry of health and family welfare	India
13	2017	Training manual for NCD programme managers at state and district level	Ministry of health and family Welfare	India
14	2017	Hypertension screening, diagnosis, assessment, and management of primary hypertension in adults in India	Ministry of health and family welfare	India
15	2017	Handbook for Counsellors - reducing risk factors for NCDs	Ministry of health and family welfare	India
16	2018	ICMR guidelines for management of type 2 diabetes 2018	Indian Council for medical research (ICMR)	India
17	2019	Guidelines for prevention and management of stroke	Ministry of health and family welfare	India
18	Not mentioned	CPHC NCD solution PHC medical Officer User manual	Ministry of health and family welfare	India
19	Not mentioned	CPHC NCD solution - NCD Application ANM User manual	Ministry of health and family welfare	India
20	Not mentioned	Module for ASHA on non-communicable diseases	Ministry of health and family welfare	India
21	Not mentioned	Module for multi-purpose workers (MPW) - Female/Male on prevention, screening and control of common non-communicable diseases	Ministry of health and family welfare	India
22	Not mentioned	Training module for staff nurses on population based screening of common non-communicable diseases	National health Mission, Ministry of health and family welfare	India
23	Not mentioned	Training module for medical officers for prevention, control and population level screening of NCDs	Ministry of health and family welfare	India
24	Not mentioned	Ayushman Bharat -comprehensive primary health care through health and wellness centres	Ministry of health and family welfare	India
25	2017	Non-communicable disease control programme (Amrutham Arogyam)-Kerala, India	National health mission, Kerala	Kerala, India
26	2021	Makkalai Thedi Maruthuvam (Reaching out health services to people)	Government of Tamil Nadu	Tamil Nadu, India
27	2009	White paper for the transformation of the health system in South Africa	Department of health Republic of South Africa	South Africa
28	2012	Quality improvement – the key to providing improved quality of care	Department of health Republic of South Africa	South Africa
29	2013-2020	National mental health policy framework and strategic plan	Department of health Republic of South Africa	South Africa
30	2013	Strategic plan for the prevention and control of non-communicable diseases (2013-17)	Department of health Republic of South Africa	South Africa
31	2015	Implementation guideline of health workforce normative guides and standards for fixed primary health care facilities	Department of health Republic of South Africa	South Africa
32	2015	National health insurance for South Africa	Department of health Republic of South Africa	South Africa
33	2015-20	Strategy for the prevention and control of obesity in South Africa	Department of health Republic of South Africa	South Africa
34	2015-20	Taxation of sugar-sweetened beverages	Department of health Republic of South Africa	South Africa
35	2017	National cancer strategic framework (NCSF)	Department of health Republic of South Africa	South Africa
36	2018	Strengthening the South African health system towards an integrated and unified health system	Department of health Republic of South Africa	South Africa
37	2019	Policy framework and strategy for ward-based primary healthcare outreach teams	Department of health Republic of South Africa	South Africa
38	2019-24	National digital health strategy for South Africa	Department of health Republic of South Africa	South Africa
39	2020	2030 human resources for health strategy: Investing in the health workforce for universal health coverage	Department of health Republic of South Africa	South Africa
40	2020	Referral policy for South African health services and referral implementation guidelines	Department of health Republic of South Africa	South Africa
41	2020	National strategic plan for the prevention and control of non-communicable diseases (2020-2025)	Department of health Republic of South Africa	South Africa
42	2021	National user guide on the prevention and treatment of hypertension in adults at the PHC level	Department of health Republic of South Africa	South Africa
43	Unknown	ICSM model health service re-organization	Department of health Republic of South Africa	South Africa
44	2013	District health system management; to take care of the population: Elderly group, chronic disease group and people with disabilities^a^	Health promotion foundation (Thai health), national health Security Office (NHSO)	Thailand
45	2013	Guides to “Flat Belly” organization	Nutrition Division, department of health, Ministry of public health, Thailand	Thailand
46	2017	Diabetes prevention and control service model high blood pressure to support the operation of NCDClinic plus	Bureau of non- communicable diseases, department of disease control, Ministry of public health	Thailand
47	2017	5-Year national NCDs prevention and control plan	Bureau of non-communicable diseases, department of disease control, Thailand	Thailand
48	2017	(Proposal) prevention and control of non-communicable diseases RTG-WHO country Co-Operation strategy January 2017–December 2021	Department of disease control, Ministry of public health, Thailand	Thailand
49	2017	(Draft) Thailand healthy lifestyle strategic plan phase II 5-year non-communicable diseases prevention and control plan (2017-2021) and related action plan	Bureau of non-communicable diseases, department of disease control, Thailand	Thailand
50	2018	Manual Tobacco addiction treatment in patients’ chronic disease in Thailand	Medical professionals network for tobacco consumption control	Thailand
51	2020	Guidelines for health system development (service plan) integrated primary care system with people at the centre (integrated, people-centred primary care)^a^(Ministry of public health, 2020)	Ministry of public health, Thailand	Thailand
52	2021	Operational guidelines NCD clinic plus & Online^a^ (department of disease control, 2021)	Division of non-communicable diseases, department of disease control, Ministry of public health, Thailand	Thailand
53	Not mentioned	Handbook of integrated, people- centred health service in new normal diabetic and hypertensive clinic (for health care workers)^a^		Thailand
54	Not mentioned	(Medical practice) comprehensive service program for managing and caring for people with diabetes and related conditions in the national health Security system “practice guideline”		Thailand

^a^Google machine translated title from original in Thai language.

Legend: NCDs= NRHM=National Rural Health Mission (India), ICMR= Indian Council of Medical Research, non-communicable disease, TB=Tuberculosis, CPHC= Comprehensive Primary Healthcare, PHC=Primary health centre, ANM=Auxiliary Nurse Midwife, ASHA=Accredited Social Health Activist, ICSM=Integrated Clinical Service Management (South Africa).

There were no policy documents specifically for NCD multi-morbidity. However, identified documents from the selected three countries addressed a few long-term NCDs and had policy directions for managing them (Online Supplemental Table S1). Policy documents did consider co-morbidities diabetes and hypertension, and guidance for health care providers (HCP) to screen or manage selected co-morbidities. For example, bi-directional screening for tuberculosis and diabetes has been recommended in India.^55^ However, none of the policies acknowledged or defined NCD multi-morbidity holistically.

While no policies explicitly acknowledged NCD multi-morbidity, there were findings from the policy documents that may be considered as potential strategies or policy actions that can be utilised to build an NCD multi-morbidity focus. The findings from the policy documents for a few NCDs have been presented under the three themes that helped to describe and highlight the gaps in policies: patient-level policy actions, healthcare provider-level policy actions, and health system-level policy actions (Table 3 and Online Supplement Table S2). Macro-level policy actions that have indirectly attempted to improve social determinants are interspersed within the appropriate themes.

**Table 3.** Summary of policy actions at patient, health care provider and health system levels.

Focus area	Actions	Description
Patient-level	Health information provision for individual NCDs	Focused on health information provision for individual NCDs like diabetes, rather than comprehensive support for multi-morbidity. Activities include campaigns, leaflets, and community health worker delivered health information provisions
	Assisted self-management support	Community health workers providing home monitoring of blood pressure and glucose, alongside education and counselling on healthy lifestyles for diabetes and hypertension
	Shared risk factor reduction	Actions aimed at reducing tobacco consumption, improving dietary practices, and enhancing physical activity
	Multi-sectoral action plans	Actions that promote inter-organisational cooperation for strategies to reduce risk factors such as collaboration with urban/transport departments to support physical activity
Health-care provider	Provider-level training and capacity building for NCD management	Training health care workers to screen for high blood pressure and blood glucose levels
	Operational guidance for improving NCD care delivery through community-level roles and coordination	Guidance on organising and planning care for NCDs. Guidance on screening and management activities such as targeted high-risk group screening, dedicated NCD case managers to coordinate services within and outside hospitals
	Enhancing human resources for healthcare	Actions focusing on improving human resource allocation based on needs, retention of trained health care staff and training of health care cadres on management of chronic conditions
Health care system	Strengthening primary care and reorganising NCD care delivery	Primary care strengthening to address growing NCDs through infrastructure, medicine availability, dedicated staff trained in NCDs and provision of electronic health records
	Reorganising primary care through integrated models to manage both NCDs and communicable diseases	Actions for managing chronic conditions, including mental health, epilepsy, asthma, hypertension, diabetes, COPD, HIV/AIDS, and TB by offering services for NCDs in clinics for HIV or TB.

#### Patient-level policy actions

At the patient-level, most activities in the policy documents were targeted at health information provision for individual NCDs such as diabetes rather than comprehensive and patient-centred self-management support aimed at NCD multi-morbidity. These health information/promotion activities included health care campaigns, distribution of patient information leaflets, and healthcare awareness through community health workers.^41,45,53,67,70,71,74,78,82,84–86,94^ South African NCD policy suggested an “assisted self-management support” where the community health care worker provides home monitoring of blood pressure and blood glucose in addition to education and counselling on healthy lifestyle behaviours intended to reduce risk factors such as overweight, tobacco cessation, etc., improving self-monitoring and medication adherence predominantly for diabetes and hypertension management.^78,82^ A similar programme called “Medicine at People’s Doorstep” or “Makkalai Thedi Maruthuvam” (MTM) was identified in Tamil Nadu, a state in India. This programme focused on home-based care and involved the delivery of drugs directly to the doorsteps of individuals aged 45 years and above who have hypertension and diabetes, as well as those with restricted or poor mobility.^67^

We identified policy documents from all three selected countries that focused on the shared risk factors and the preventive measures related to NCDs, such as policies on reducing tobacco consumption, better dietary practices, and improving physical activity.^44,46,47,49–51,53,56,57,59,62,63,65,66,75,82,87,89,91,92^ South Africa had a policy dedicated to reducing obesity.^74^ Similarly, we identified a strategy for improving physical activity in Thailand, which the WHO acknowledged as an essential step towards promoting physical activity.^96^ Furthermore, all three selected countries recognised the need for a multi-sectoral approach for health promotional activities related to NCDs in their policy documents. For example, the National Action Plan and Monitoring framework for preventing and controlling NCDs^48^ in India recognised the need to work with the urban department or transport department to create and preserve environments for supporting physical activity. Policies on reducing risk factors acknowledged poverty, lack of access for physical activity, and lack of social support as drivers for obesity and increasing tobacco consumption. For example, South African policy for reducing obesity has acknowledged poverty as a driver for obesity.^74^ However, the policy actions have been directed at improving awareness and suggestions for improving multi-sectoral co-ordination.

#### Healthcare provider-level policy actions

Most policy documents identified from the selected three countries for NCDs involved guidance or standards for healthcare providers and operational guidance for the main organisation-level activities to improve NCD care delivery. Of those policy documents from India addressing NCD management, provider-level activities pertained mainly to training and capacity building.^46–51,56–63,65,73,80,83,85,90–92,95^ We identified general guidance documents for training and actions to be carried out at the primary care level by the different cadres of health care providers from all three selected countries. For example, in India, the identified policy documents detailed guidance for training ASHAs (Accredited Social Health Activists) in screening for high blood pressure and blood glucose levels.^50,62^ Similarly, the role of village health volunteers in monitoring the self-measurement of blood pressure in high-risk groups was detailed in documents identified from Thailand. The use of NCD case managers of diabetes/hypertension clinics to coordinate and link services inside and outside hospitals were recommended in policy documents from Thailand.^85^ In South Africa, NCD policy recommended training of public health nurses on chronic conditions and community health workers for community level support.^77,82,84^ Furthermore, a policy document on improving the human resources for healthcare based on current and future needs was identified from South Africa.^80^

#### Health system level actions

At the health system or organisational level, policy documents detailing the directions to reorganise care for people with NCDs, strengthen primary care, and achieve universal health coverage have been identified from all three selected countries.^57,66,72,77,78,82,84,93,94^ However, the reorganisation focus was primarily on provisions of physical infrastructure, medicine availability at primary care levels, and incorporation of patient medical records at the facility level. Additionally, a focus on providing quality care was emphasised in some documents. For example, under the National Programme for Prevention & Control of Cancer, Diabetes, Cardiovascular Diseases & Stroke (NPCDCS), different states in India initiated various programmes to control and manage chronic diseases and their complications.^41,45,48^ The policy documents from the state of Kerala in India enabled actions to strengthen the health system (primary care) by establishing family health centres with enhanced infrastructure, availability of medicines for common chronic NCDs, and electronic health records for all patients.^66^ Furthermore, under the Ayushman Bharat programme (Government of India), the creation of health and wellness centres staffed with mid-level health providers was recommended for better prevention and coverage of NCDs.^57^Similarly in South Africa, a primary care reorganisation and strengthening initiative for managing chronic conditions was introduced as a pilot through the Integrated Chronic Disease Management (ICDM) model in 2011. The chronic diseases covered under the ICDM model include NCDs such as mental health conditions, epilepsy, asthma, hypertension, diabetes, and chronic obstructive pulmonary disease (COPD). Additionally, the model also addresses communicable diseases, encompassing HIV/AIDS and all forms of tuberculosis (TB). This initiative was further expanded to cover all primary care health services through Integrated Clinical Services Model.^82,84^

## Discussion

We examined 54 current policies and guidelines from India, Thailand, and South Africa to identify how policies acknowledged and suggested policy directions for managing NCD multi-morbidity. We employed an analytical framework to review and organise the content of policies and guidelines available online. We found that all three countries addressed the management of NCDs, particularly diabetes and hypertension, at the primary care level and focused on strengthening and reorganising primary care for managing these conditions. Furthermore, all three countries had policies for addressing shared risk factors, such as tobacco use and physical inactivity, which are likely to impact the prevention and management of NCD multi-morbidity. However, certain differences also exist among the countries in their focus on risk factors. For instance, South Africa has a dedicated policy aimed at reducing obesity, Thailand adopts a targeted approach to address obesity reduction among the working population, and India emphasises an overall risk reduction approach for NCDs. Additionally, the policies from the selected three countries did not address any explicit improvement in social determinants of health to aid in the prevention and control efforts of NCD multi-morbidity.

None of the current policy review documents acknowledged NCD multi-morbidity beyond the co-occurrence of diabetes and hypertension. In a similar policy review comparing policies for multi-morbidity in the UK, Australia, and Sri Lanka, only the UK had a dedicated policy for multi-morbidity.^97^ One significant implication of the absence of a dedicated policy is the failure to recognise the significance of multi-morbidity as an issue. However, this is not to suggest that the presence of an approach would result in better outcomes, as previous studies looking at NCDs and health system preparedness found no association between policy and high-quality care outcomes.^98–100^ Considering that many LMICs are experiencing an epidemiological transition and grappling with chronic infectious diseases and NCDs, it was expected that there would be an acknowledgment of the coexistence of NCD multi-morbidity and a need for coordinated management efforts in its policy documents.

Without clear policies and management plans, several potential drawbacks may arise in NCD multi-morbidity prevention and control efforts in LMICs. Previous studies examining patient experiences with NCD multi-morbidity identified the increased workload involved, encompassing self-care management, intricate treatment modalities, behaviour change, and multiple clinic visits as barriers to effective care.^16,17^ Patients with multi-morbidity often face diminished capacity, including compromised physical and mental functioning, limited health literacy, and insufficient family and social support, resulting in suboptimal outcomes.^17,20^ Moreover, multi-morbidity is more prevalent among individuals from lower socioeconomic backgrounds, who frequently encounter resource constraints in effectively managing their health issues.^101–103^ Consequently, policies targeting multi-morbidity management in LMICs must account for the unique challenges patients face in these settings and formulate interventions that specifically address these challenges.^104^

Recognition of the need for self-management was evident in the NCD policies of all three countries. However, limited consideration was given to the complexities of self-management for patients with NCD multi-morbidity in these contexts, resulting in minimal tailored support. The United Kingdom NICE (National Institute for Health and Care Excellence) guidelines suggest that policy actions in multi-morbidity should strive to empower patients and facilitate self-management.^105^ Potential interventions could encompass digital reminders for medication and appointments, doorstep availability of medicines for elderly patients with chronic NCDs, outreach programs, and educational initiatives for improving awareness and tailored support for self-management.^106–109^

Strategies at the Healthcare provider level should include technical training and capacity building for healthcare workers.^110^ A shift towards an integrated generalist approach to healthcare is crucial, moving away from an excessive emphasis on specialised roles based on a country’s context and capacity. Policy actions should address human resource shortages and integrate frontline health workers into the formal healthcare system.^111^ Longer consultation times are necessary for patients with multi-morbidity, especially in deprived regions where individuals face higher mortality risks and a range of chronic conditions. If expanded beyond specific NCDs, Thailand’s case manager model could be a suitable framework for future management of NCD multi-morbidities.^92^Task-sharing strategies involving increased participation of health workers may enhance communication and provide tailored self-management support.^112^

At the organisation level, there is a need to transition towards designing better-integrated systems encompassing primary and secondary care, social support systems, and mental healthcare to reduce fragmentation of care.^113,114^ Developing an integrated system requires attention to workforce planning in LMIC settings.^8,113^ Integrated clinical algorithms, standardised clinic stationery, and reorganised clinic flows may improve care delivery efficiency for managing NCD multi-morbidity.^36^ Previous studies from South Africa have shown that better staff support and leadership is needed for integrated models to be sustainable.^115–118^ Strategies to enhance the skills among generalist healthcare professionals and the establishment of integrated chronic disease teams to provide minimally disruptive care to individuals with multi-morbidity should be prioritised in primary care settings.

One of the overarching findings from the analysis is that all three countries have implemented policy measures to prevent various risk factors associated with NCDs. To enhance the comprehensiveness of these policies, it could be valuable to include specific components that acknowledge shared risk factors, particularly behavioural determinants contributing to NCD multi-morbidity.^12^ For instance, obesity increases the likelihood of both osteoarthritis and type 2 diabetes.^119^ Specific policies targeting shared risk factors, such as policies on physical activity in Thailand and obesity prevention policies in South Africa, may be adopted for India and other LMICs.^45–47^ Preventing multi-morbidity in LMICs requires population-level structural changes in policies that go beyond individual behaviour change strategies, addressing risk factors within the specific socioeconomic-political context, including interventions targeting the food system and built environment to reduce socioeconomic inequalities.^120,121^

At the policy level, there is a notable absence of recognition regarding the coexistence of multiple NCDs and their adverse interactions. Such negative interactions may even affect the health and well-being of the affected individual’s family members with multi-morbidity. Social determinants of health play a vital role in such clustering of chronic conditions and their interactions.^122^ To adopt a comprehensive perspective, it is necessary to move beyond narrow approaches such as the co-occurrence of conditions and develop an all-inclusive understanding of disease clustering, interactions, and their biological, ecological, and social contexts. Ultimately, this approach calls for comprehensive public health policies that address the underlying causes of diseases.^123^

## Limitations

Our search may have missed policy documents that are not publicly available and in regional languages. While we engaged with experts from each country to gather relevant documents, we might have missed some. Second, we have considered only policy content analysis rather than the health care system implementation and delivery in each country. This review is a first step in identifying and highlighting the lack of policies dedicated to multi-morbidity in LMICs.

## Conclusion

The research examined the current policies, approaches, and deficiencies for people with multiple long-term conditions in three LMICs. The primary results indicate that all the selected three countries prioritise preventative measures such as screening, identifying risk factors, and promoting healthy behaviours to combat NCDs. Nevertheless, they fall short in acknowledging the impact of NCD multi-morbidity and its effective management in primary care settings. There is a lack of appreciation of the social determinants of health and strategies to tackle them to prevent and control multi-morbidity in the existing policies.

## Supplemental Material

Supplemental Material - Non-communicable disease multi-morbidity in policies from India, Thailand, and South Africa: A comparative document review Supplemental Material for Non-communicable disease multi-morbidity in policies from India, Thailand, and South Africa: A comparative document review by Linju Joseph, Rakhal Gaitonde, Charutha Retnakumar, Athira Krishnan, Thoniparambil Ravindranathanpillai Lekha, Neethu Sasidharan, André van Rensburg, Naomi Levitt, Nilawan Upakdee, Jissa Vinoda Thulaseedharan Mathew Joseph Valamparampil, Sivadasanpillai Harikrishnan, Sheila Greenfield, Paramjit Gill, Justine Davies, Semira Manaseki-Holland and Panniyammakal Jeemon in Journal of Multimorbidity and Comorbidity

## Data Availability

All relevant data are included in this manuscript. Data may be shared upon a reasonable request and is provided to the corresponding author. *

## Appendix

### List of Abbreviations

NCD: Non-Communicable Diseases
LMIC: Low- and Middle-Income Countries
WHO: World Health Organization
