# Supplementary material for: Non-communicable disease multi-morbidity in policies from India, Thailand, and South Africa: A comparative document review
Source: J Multimorb Comorb. 2025 Apr 13;15:26335565251330371. doi: 10.1177/26335565251330371 (PMC12033547; doi:10.1177/26335565251330371)
Supplement: Supplemental Material - Non-communicable disease multi-morbidity in policies from India, Thailand, and South Africa: A comparative document review [file sj-pdf-1-cob-10.1177_26335565251330371.pdf]

Online supplement: Table S1 Summary of included policy documents

No.	Name of document	Year	Publication	Strategy of the policy and area covered	Summary
1.	2030 Human Resources for Health Strategy: Investing in the Health Workforce for Universal Health Coverage	2020	Department of Health Republic of South Africa	Health workforce planning for improving quality care<br><br>Health system level<br><br>Universal health coverage<br><br>Comprehensive and integrated care	• Align health workforce planning with present and future demands.<br>• Foster a multi-disciplinary, compassionate healthcare workforce.<br>• Promote equity and social responsibility in education and training.<br>• Institutionalize data-driven and research-informed policies.<br>• Utilize evidence-based approaches for workforce management.<br>• Ensure investments in healthcare personnel are well informed and effective.<br>• Strive for comprehensive health workforce strategies.
2.	National Health Insurance For South Africa	2015	Department of Health Republic of South Africa	Health system level<br><br>Ensure quality and affordable health services irrespective of socioeconomic status.<br><br>Massive reorganisation of the private and public health system<br><br>Public health system: According to the NHI policy, the' heartbeat of the public health system is primary health care,<br><br>Suggests an ideal clinic concept.<br><br>Comprehensive and integrated care	• National Health Insurance (NHI) aims to achieve universal health coverage.<br>• NHI will expand healthcare access to a broader population.<br>• It seeks to enhance both the quality and quantity of available healthcare services.<br>• The goal is to reduce direct healthcare costs for covered individuals.<br>• NHI's objective is to shield people from out-of-pocket expenses.<br>• It aims to prevent financial crises linked to healthcare expenses
3	White Paper for The	2009	Department of Health	Health system-level	• Reorganizing the department of health

	Transformation Of The Health System In South Africa		Republic of South Africa	comprehensive and integrated health services<br><br>Restructuring of the health sector.<br><br>Not particularly for NCD/multi-morbidity/ chronic condition<br><br>Comprehensive and integrated care	• Restructuring of provincial and district health<br>• Integrating the public and private sectors<br>• Community involvement- people should be equipped with the information and the means for identifying behavioural change conducive to improvement in their health<br>• Restructuring of primary health care
4	National user guide on the prevention and treatment of hypertension in adults at the PHC level	2021	Department of Health Republic of South Africa	Health system related<br><br>Mention the definition of co-morbidities<br><br>Risk factors of hypertension<br><br>Screening and early detection<br><br>Alternate medicine management if there are other co-morbidities.<br><br>Policies on treatment regimens for co-morbidities.	• This user guide aims to intentionally address the detection and management of hypertension in order to prevent and mitigate hypertension against the detrimental effects on each individual.<br>• It is a user guide for Primary Health Care professionals to screen, detect and appropriately manage, monitor and refer if necessary. It also includes hypertension management in special populations such as those with diabetes, pregnancy, HIV/AIDS, known existing cardiovascular conditions and children and adolescents.

5	National Cancer Strategic Framework (NCSF)	2017	Department of Health Republic of South Africa	Multi-sectoral approach for cancer prevention and control<br><br>enactment of tobacco control legislation like the Tobacco Products<br><br>Risk factors<br><br>Early screening and treatment<br><br>Rehabilitation<br><br>Inter-sectoral approach<br>Macro-level interventions.	• To reduce cancer burden and promote patient–centred and human rights-based care through integrated and evidence-based programs on cancer surveillance, prevention, early detection, screening diagnosis, treatment, rehabilitation and support, and palliative care.<br>• Create a multi-sector, inter-program, coordinated response to cancer prevention and control at national and provincial levels.
6	National Mental Health Policy Framework and Strategic Plan	2013-2020	Department of Health Republic of South Africa	Association of Mental Health disorders and other illnesses<br><br>address various biological, psychological and social determinants of mental illness<br><br>Integrated care<br><br>Inter-sectoral collaboration<br><br>Organisation of service<br><br>Psychological support	• Expand decentralized primary mental health services, including community-based, clinic, and hospital care.<br>• Increase public awareness and combat stigma associated with mental illness.<br>• Promote collaboration between the Department of Health and other sectors to protect human rights and enhance mental health.<br>• Empower local communities, especially service users and caregivers, to promote mental well-being and recovery.<br>• Adopt a multi-sectoral approach to address the link between poverty and mental ill-health.<br>• Establish a monitoring and evaluation system for evidence-based mental health services.<br>• These strategies aim to improve mental healthcare accessibility, reduce stigma,

					empower communities, address socio-economic factors, and ensure evidence-based practices in South Africa's mental health system.
7.	Referral Policy for South African Health Services and Referral Implementation Guidelines	2020	Department of Health Republic of South Africa	Health system related<br><br>Referral practices<br><br>Comprehensive and integrated care	• Ensure that clients receive optimal care at the appropriate level.<br>• Strengthen coordination and complementation between levels of care, units and institutions in caring for clients.<br>• Facilitate optimal and cost-effective use of high specialist facilities such as hospitals by patients within and across provincial borders.<br>• Strengthen peripheral health facilities through feedback that will enhance the skills of the referring doctors by confirming or not confirming diagnoses.<br>• Enhance utilization of services at the PHC level by those in need of care.<br>• Progressively reduce any unnecessary burden on tertiary and specialist hospitals.<br>• Define governance and other arrangements for referrals between public and private sectors.
8	National Digital Health Strategy for South Africa	2019-24	Department of Health Republic of South Africa	Health system related<br><br>Complete health electronic record<br><br>Digitalisation of health system<br><br>Establish an integrated platform for health sector information system<br><br>Scale up high impact mhealth for community-based interventions.	Priorities for digital health-<br>• a person-centred approach considers all citizens as participants that either require or provide care that is preventative, curative, palliative, or a combination at multiple intervals between two major life events, birth, and death.<br>• Helps to establish an integrated platform for health sector information systems.

9	Implementation Guideline of Health Workforce Normative Guides and Standards for Fixed Primary Health Care Facilities	2015	Department of Health Republic of South Africa	Health system related<br><br>Equitable distribution of health workforce based on normative guides<br><br>Comprehensive and integrated care	• Guidelines related to the distribution of work force in the health care delivery system
10	Strategy for the prevention and control of obesity in South Africa	2015-20	Department of Health Republic of South Africa	Population-based approach<br><br>System based perspective<br><br>Inter-sectoral engagement<br><br>multidisciplinary, multi-sectoral coordinating structure<br><br>Mobilization of key stakeholders<br><br>Inter-sectoral approach	• Create an institutional framework to support inter-sectoral engagement and an enabling environment that supports the availability and accessibility of healthy food choices in various settings for the prevention and control of obesity.<br>• Communicate with, educate and mobilise communities and increase the percentage of the population engaging in physical activity<br>• Support obesity prevention in early childhood (in-utero – 12 years), establish a surveillance system and strengthen monitoring, evaluation, and research.
11	National Strategic Plan for The Prevention And Control Of Non-Communicable Diseases 2020-2025	2020	Department of Health Republic of South Africa	Policy related to NCD/chronic long-term condition<br><br>Health promotion<br><br>Air pollution<br><br>Comprehensive and integrated approach<br><br>District multi-sectoral coordination<br><br>Capacity building<br><br>Identification of strategic action areas<br><br>Governance	• Integrated care, prevention of behavioural risk factors for NCDs, smoking has negative impacts on all the major NCDs including lung and other cancers, chronic obstructive pulmonary diseases (COPD), heart disease, stroke, and diabetes.<br>• Integrated Clinical Services Model that incorporates all chronic diseases (whether communicable or non-communicable, as a part of the Ideal Clinic initiative), integration of NCDs in the Primary Health Care Service Package (2015) (incorporating most common NCDs (including Asthma/COPD; Cardiovascular Disease, Diabetes, Mental Health conditions; Epilepsy, Musculoskeletal Disorders)

					• Centralized Chronic Medicines Dispensing and Distribution (CCMDD) model for distribution of medicine.<br>• Promote healthy diet high in fruits and vegetables and low in saturated fat/trans-fat, free sugar and salt.<br>Promote physical activity
12	ICSM model Health Service Re-organization	Unknow n	Department of Health Republic of South Africa	Health service reorganisation<br><br>Management support<br><br>Assisted self-management using Ward-based primary healthcare outreach team<br><br>Awareness and screening<br><br>Health system strengthening<br><br>Self-management	• Health service re-organisation envisages clinical management support, assisted self-management, population health awareness and screening, single administrative point, pre-appointment retrieval of clinical records, clinical guidelines and tools, health promotion, education at community level and identification of at-risk patients.
13	Taxation Of Sugar-Sweetened Beverages	2015-20	Department of Health Republic of South Africa	Mentioned non-communicable diseases<br><br>Macro-level intervention-taxation of sugar-sweetened beverages<br><br>Inter-sectoral approach	For the prevention and control of obesity, the overarching goals are as follows:<br><br>• Create an institutional framework to support inter-sectoral engagement.<br>• Create an enabling environment that supports the availability and accessibility of healthy food choices in various settings.<br>• Increase the percentage of the population engaging in physical activity.<br>• Support obesity prevention in early childhood (in-utero – 12 years).<br>• Communicate with, educate and mobilize communities.<br>• Establish a surveillance system, strengthen monitoring and evaluation, and research.
14	Strategic Plan for The	2013	Department of Health Republic of South	Mentioned non-communicable diseases	• Raise taxes on tobacco products and alcoholic beverages

	Prevention And Control Of Non-Communicable Diseases 2013-17		Africa	and their major risk factors<br><br>Population, community and individual level interventions.<br><br>Health system strengthening and reform<br><br>Multi-sectoral approach<br><br>Innovative research<br><br>Screening<br><br>Provider level intervention<br><br>Policies related to multiple chronic conditions/co-morbidities	• Enforce bans on tobacco and alcoholic advertising<br>• Smoke free workplaces<br>• Restrict access to retiled alcohol<br>• Enforce drink-driving laws<br>Reduce salt intake<br>Enforce food taxes on unhealthy food (foods high in fats and sugar) and food subsidies on healthy food (fruits and vegetables)<br>• Physician counselling<br>• Cervical Cancer Screening (PAP Smear) and treatment<br>Inter-sectoral Collaboration for Prevention of NCDs
15	Quality Improvement – the key to providing improved quality of care	2012	Department of Health Republic of South Africa	Quality improvement across all health establishment<br><br>Health system level	• Develop a common definition of quality of care in all health establishments as a guide for the public, managers and all health care workers.<br>• Establish a national benchmark against which health establishments can be assessed and provide a framework for the certification of health establishments.<br>• Provide a common tool to identify gaps, appraise strengths and guide quality improvement for care.
16	Policy Framework and Strategy for Ward-Based Primary Healthcare Outreach Teams	2018	Department of Health Republic of South Africa	Health system level<br><br>Community participation<br><br>Inter-sectoral collaboration<br><br>Context-specific implementation<br><br>transparency<br><br>Equitable distribution of community	• An equitable distribution of a comprehensive community-based PHC service that will contribute to the improvement of health and well-being of individuals, households and communities being served.

				services through ward-based primary health care outreach team<br><br>Health system level	
17	Strengthening the South African health system towards an integrated and unified health system	2018	Department of Health Republic of South Africa	Improving the public healthcare system and thereby increasing the accessibility of quality healthcare.<br><br>Health system level	• The implementation of this compact is expected to contribute significantly to improving the public healthcare system so that many more South Africans can access quality healthcare.<br>• The participation of the government and the critical stakeholders in the development and implementation of various components of the compact will deliver significant benefits to the health system, thereby making a significant contribution to the realization of the Constitutional right to Health through enhancing South Africa's economic and social potential; and enabling people to live healthy and productive lives.
	THAILAND				
1	District health system management; To take care of the population: Elderly group, Chronic disease group and People with disabilities	2013	Health Promotion Foundation (Thai Health), National Health Security Office (NHSO)	Patient –oriented services<br><br>Disease prevention<br><br>Health promotion, maintenance and rehabilitation.<br><br>Integrated care model.<br><br>Health promotion.	• This document pertains to the health care services with emphasis on population health, addressing common needs and shared risks within specific populations.<br>• Primary care services should be designed based on the collective needs or risks, with individual considerations.<br>• Healthcare services should encompass health promotion, disease prevention, maintenance, and rehabilitation.<br>• This document focuses on three key population groups: the elderly, chronic disease patients, and individuals with disabilities, highlighting their significance in public health.
2	Guides to 'Flat belly'	2013	Nutrition Division,	Obesity control	• Any community or organisation having people

	organisation		Department of health, Ministry of public health.	Risk factor reduction<br><br>Macro-level intervention	with fat belly will decrease working capacity and reduce productivity. Therefore, the Department of health, Ministry of public health in coordination with Thai health promotion foundation (Thai health) introduced Flat belly organisation under the protect 'Partnership for flat belly of Thai people'.<br>• It aims to encourage organisations to participate in reducing belly fat within their organisations and the manual provides guidelines in this context.<br>• It intends to promote and enhance the knowledge awareness and skills of the organisations to reduce obesity or fat belly of people in their organisation.<br>• The guide also focuses on prevention and control of fat belly in a group of normal people by using the 3-E concept; Eating, Exercise and Emotion.
3	Diabetes Prevention and Control Service Model high blood pressure<br>To support the operation of NCD Clinic Plus	2017	Bureau of Non-Communicable Diseases, Department of Disease Control, Ministry of Public Health	Self-management<br><br>Early detection and screening	• This document serves as a guide for preventing and controlling diabetes and hypertension at the community level.<br>• It outlines principles for screening high blood glucose and blood pressure and delivering preventive services.<br>• It recommends motivational interviewing to encourage behaviour change, empowering patients with self-care knowledge and skills.<br>• It highlights community-based activities aimed at preventing diabetes and hypertension by promoting behavioural changes.

4	5-Year National NCDs Prevention and Control Plan (2017-2021)	2017-2021	The Policy and Strategy Section, Bureau of Non-Communicable Disease, Ministry of Public Health.	Inter- sectoral approach.<br>Policies and laws supporting NCD prevention and control.<br><br>Inter- sectoral approach.	• This document aims to respond to the disease prevention and control for NCDs. Specifically, to achieve 9 global goals, which are adjusted to align with the situation of Thailand and the No. 12 National Economic and Social Development Plan and the 20-year National Strategic Plan (in relation to public health).<br>• The content of the Plan focuses on creating participation of population, communities, local administrations, and various sectors, improving the operating efficiency with<br>the use of data and enhancing potential of people to enable self-care of their health.<br>• Moreover, it emphasizes on integration with other strategic plans and avoid redundancy with other related national strategic plans as well as NCDs situation analysis and the situation of NCDs Implementation system in the past 5 years.
5	(Proposal) Prevention and Control of Non-communicable Diseases RTG-WHO Country Co-operation Strategy. January 2017—December 2021	2017-2021		Inter-sectoral coordination<br><br>System-policy level<br><br>Quality care services<br><br>Surveillance<br><br>NCD risk factor reduction<br><br>Inter-sectoral approach	• To tackle NCDs and risk factors among Thai population, national NCD strategic plan was developed based on the Thai healthy lifestyle strategy.<br>• The strategy adopts nine national targets in line with the global targets with a focus on “systems improvement” using existing formal and informal networks and engaging multiple stakeholders from government agencies as well as civil society.<br>• The programme will provide strategic support and catalyse the implementation of the national NCD strategic plan and other existing strategies for NCD risk factors.<br>• Building upon the previous CCS, the new programme aims to facilitate multi-stakeholder co-ordination, supports knowledge generation

					and dissemination networks, strengthen surveillance, monitoring and accountability system, and facilitate international cooperation to drive the global NCD movement.<br>• The programmes also aim to improve tobacco and alcohol control policies, improve policies to reduce obesity including unhealthy diet and physical inactivity and to strengthen the quality of hypertension and diabetes services and capacity building of stakeholders.
6	(Draft) Thailand Healthy Lifestyle Strategic Plan Phase II<br>5-Year Non-Communicable Diseases Prevention and Control Plan (2017-2021) and related Action Plan	2017-2021		Risk factors<br>Intersectoral coordination<br>System level policy<br>Community participation<br>Intersectoral coordination	• The plan focuses on relieving the avoidable burden of sickness, death, and disability due to non-communicable diseases by minimizing risk factors through promoting community participation, emphasising on self-management, intersectoral coordination and strengthening international collaboration and joint policy-driven progress.<br>• To promote wellbeing of people and maximize productivity of all age groups and to prevent these non-communicable diseases from obstructing the quality of life.
7	Manual 1<br>Tobacco addiction treatment in patients' chronic disease in Thailand	2018	Manual of Medicine	Smoking in chronic disease patients<br>Tobacco control, cessation, treatment.<br>Treatment	• The manual provides guideline for managing chronic disease patients with smoking.<br>• It recommends the hospitals to develop a treatment and referral system to treat patients for quitting tobacco quickly which will help prevent complications of tobacco use to various chronic diseases.<br>• To ensure that all tobacco users receive treatment as soon as possible to have an impact on life and health.<br>• It envisages that the goal of treating patients is to completely stop taking the drug, not

					accepting to reduce the amount of use without cessation.
8	Guidelines for health system development (Service Plan)<br>Integrated primary care system with people at the centre (Integrated, People-centred Primary Care)<br>ISBN: 978-616-11-4241-4	2020	Ministry of Public Health	Health system level changes including shared care plan, patient registry.<br>Family care team for elderly, terminally ill, handicapped and patients with chronic conditions.<br>Promotion of Thai traditional medicines.<br><br>Integrated primary health care services.<br>Health system level changes<br>Self-management	• This operational guideline is the result of the service model development with the cooperation of the Ministry Public Health Faculty of Medicine Ramathibodi Hospital Mahidol University and a network of primary care units in the area by focusing on changing the relationship to be closer to the people focus on health promotion and health knowledge.<br>• To increase the ability to manage their own health concretely.<br>• There are clear guidelines for changing primary health systems which will be useful for health teams, agencies or related organisations in applying them to create learning and develop primary care units and networks.
9	Operational Guidelines<br>NCD Clinic Plus & Online.	2021	Division of Non-Communicable Diseases, Department of Disease Control, Ministry of Public Health.	Quality assessment of NCD prevention and control services of public health facilities.<br>Health system level<br>Quality assessment of NCD prevention and control services of public health facilities.	• The guide line consists of a 6-component procedural assessment and an outcome assessment.<br>• It is like a self-assessment to improve the quality of NCD prevention and control services of public health facilities.<br>• Assessment of service indicator outcomes that reflect processes and outcomes for prevention and control of NCDs and its complications consisted of indicators for DM, HT, CVD and CKD.<br>• Recommends screening for diabetes and high blood pressure according to standard guidelines and register separately as normal groups, high risk groups and sick groups. It also recommends to measure the evaluation results

					to improve the service quality of NCD clinics comprehensively
10	Handbook of integrated, people-centred health service in new normal<br>diabetic and hypertensive clinic (for health care workers) *	Not mention ed		Empowerment and community engagement.<br>Risk stratification.<br>Re-orienting care model.<br>Comprehensive care	• Aims at empowering people and communities, strengthen system governance and building people-centred approach in health systems introducing integrated health services.<br>• Emphasises the need for building self-management abilities in patients to manage their condition. Both patients and service providers has their role in creating this knowledge.
11	(medical practice) Comprehensive service program for managing and caring for people with diabetes and related conditions in the National Health Security System<br>"Practice Guideline"	Not mention ed		Screening<br>Treatment monitoring and evaluation<br>Health education<br>Comprehensive care	• The burden of diabetes among Thai population could not just be addressed with educating people rather including other social and behavioural factors.<br>• The program provides guidelines for early detection, risk management as well as monitoring and evaluation.
	INDIA				
1	National Programme for Prevention and Control of Cancer, Diabetes, Cardiovascular Diseases and Stroke (Ministry of Health and Family Welfare, 2010)	2010,2013 revised	Ministry of Health and Family Welfare	Policies related to multiple chronic conditions such as cancer, cardiovascular diseases, stroke, diabetes and hypertension<br>Health system level	• Focus on strengthening infrastructure, human resource development, health promotion, early diagnosis, management and referral.
2	Operational	2011	NRHM, Ministry of	Elderly care- Preventive, curative and	• The programme (NPHCE) attempts to

	guidelines-National programme for health care of the elderly (Ministry of Health and Family Welfare, 2011)		Health and Family Welfare	rehabilitative<br>Comprehensive care	introduce a comprehensive health care set up completely dedicated and tuned to the needs of the elderly.<br>• The interventions are designed to capture the preventive, curative and rehabilitative aspects in the geriatric field.
3	Twelfth Five Year Plan (2012–2017)	2012	Planning Commission, Government of India	Inter-sectoral approach<br>Health system level	• Policy interventions like raising taxes on tobacco, enforcing bans on tobacco consumption in electronic media, counselling for quitting tobacco, early detection and effective control of high blood pressure and diabetes, screening for common and treatable cancers; and salt reduction in processed foods will be taken up for NCDs.<br>• Institutions like schools, workplaces and prisons provide opportunities for preventive health check-ups, regular and group exercises, early detection of disease and for dissemination of information on lifestyle choices, yoga, exercise and healthy living.<br>• Employees and workers will be informed of the ill-effects of sedentary lifestyle, and encouraged to increase physical activity.
4	National Action Plan and Monitoring Framework for Prevention and control of NCDs	2012	Ministry of Health and Family Welfare	Framework with indicators and targets and suggested action points by diverse sectors (schools, work places etc.) Implement strategies under NPCDCS<br>- Inter-sectoral approach	• Health promotion and information, education, communication programmes, promotion of physical activities, diet modification and other lifestyle modifications.<br>• Early detection and treatment of NCD.<br>• Multi-sectoral approach involving urban development, department/municipal corporation/transport department etc. for creation and preservation of environments supporting physical activities in community setting, e.g. development of safe and accessible infrastructure for walking and cycling

5	Standard treatment guidelines- Hypertension Screening, Diagnosis, Assessment, and Management of Primary Hypertension in Adults in India (Ministry of Health and Family Welfare,2016a)	2016	Ministry of Health and Family Welfare	Comprehensive care	• The guideline has a primary care focus and a public health approach involving 5 components namely prevention, screening, diagnosis, management including lifestyle changes, follow-up and monitoring adherence.
6	Operational Framework Management of Common Cancers(Ministry of Health and Family Welfare, 2016b)	2016	Ministry of Health and Family Welfare	Screening and early detection	• Provides feasible strategies in early detection of common cancers namely breast, cervical and oral cancers including referral and treatment services, required resources, training, behavioural change communication, monitoring and evaluation
7	Operational Guidelines on Prevention, Screening and Control of Common NCDs (Ministry of Health and Family Welfare, 2016c)	2016	Ministry of Health and Family Welfare	Comprehensive Primary Health Care	• Guidelines on Prevention, Screening and Control of Common NCDs- Hypertension, Diabetes, common cancers like oral, breast, cervical cancers.
8	Training Manual for Medical Officers on Reducing Risk Factors of NCDs(Ministry of Health and Family Welfare, 2016d)	2016	Ministry of Health and Family Welfare	Prevention<br>Early detection<br>Intervention<br>risk factor assessment	• It intends to make medical officers familiarise with behavioural and psychological risk factors for non-communicable diseases<br>• To provide them with the skills to identify and reduce these risks, particularly in the clinical setting.
9	Training Manual for Community Health	2016	Ministry of Health and Family Welfare	Prevention<br>Early detection	• Train the community health workers to familiarise with behavioural and psychological

	Workers on Reducing Risk Factors of NCDs(Ministry of Health and Family Welfare, 2016e)			Intervention<br>risk factor assessment	risk factors for non-communicable diseases<br>• Intends to provide them with the skills to identify and reduce these risks
10	Training Manual for NCD Programme Managers at State and District Level	2017	Ministry of Health and Family Welfare	Program implementation<br>Multisectoral approach	• This manual aimed at strengthening managerial skills and competencies of NPCDCS programme managers in implementing programme efficiently and identify the need for multi sectoral convergence to effectively control NCDs.<br>• To familiarise them with strategies for involving different stakeholders in the programme.
11	National Health Policy (Ministry of Health and Family Welfare, 2017a)	2017	Ministry of Health and Family Welfare	Universal health coverage<br>OOPE<br>Multisectoral approach	• Improve health status through concerted policy action in all sectors<br>• Expand preventive, promotive, curative, palliative and rehabilitative services provided through the public health sector with focus on quality and lowering the cost of healthcare delivery.
12	National multi-sectoral action plan for prevention and control of common non-communicable disease	2017-22	Ministry of Health and Family Welfare	The Action Plan provides a framework to support and strengthen a partnership with non-health stakeholders to integrate NCD prevention strategies within their plans and programmes. Additionally, within the health sector, the Action Plan will build synergies with the existing programs<br>Inter-sectoral approach<br><br>Health system level	• To achieve health promotion that include reduction of NCD risk factor levels in children, adolescents and adults. The risk factors include behavioural risk factors such as tobacco use, alcohol use, unhealthy diet, physical inactivity, environmental pollution including household air pollution and other social determinants.<br>• Ensure NCD related health services are incorporated under the Universal Health Coverage as part of health system strengthening.<br>• Strengthen of primary health care system to improve prevention, screening, early diagnosis, and sustained management of people with or at high risk for major NCDs in order to prevent

					complications, reduce the need for hospitalization and costly high-technology interventions and premature deaths.
13	Hypertension Screening, Diagnosis, Assessment, and Management of Primary Hypertension in Adults in India(Ministry of Health and Family Welfare, 2017c)	2017	Ministry of Health and Family Welfare	Prevention, screening, diagnosis, management including lifestyle changes and follow-up, monitoring adherence.<br>Comprehensive care	• The guideline has a primary care focus and a public health approach via five components namely prevention, screening, diagnosis, management including lifestyle changes and follow-up, monitoring adherence.
14	National Framework for Joint TB-Diabetes Collaborative Activities (Ministry of Health and Family Welfare, 2017d)	2017	Ministry of Health and Family Welfare	Management of TB and DM<br>Prevention and control of diabetes and TB	• The framework aims to guide national programmes, health personnel and others engaged in care of patients and prevention and control of diabetes and TB on how to establish a coordinated response to both diseases at the state, district and lower levels.<br>• It will guide programme managers of RNTCP and NPCDCS in reducing morbidity and mortality due to TB and diabetes through prevention, bidirectional screening for early detection and prompt management of TB and diabetes.
15	Handbook for Counsellors - Reducing Risk Factors for NCDs(Ministry of Health and Family Welfare, 2017e)	2017	Ministry of Health and Family Welfare	Behavioural change<br>Risk factor reduction<br>Risk factor reduction via behavioural change	• It aims at providing required knowledge and skills to motivate the community and NCD patients to change/initiate and maintain healthy behaviours that will ensure optimal health.
16	ICMR Guidelines for management of type 2 diabetes 2018 (ICMR, 2018)	2018	Indian Council for Medical Research (ICMR)	Management of type 2 DM and comorbid conditions like hypertension, dyslipidaemia, obesity and TB.<br>Comorbidity management	• It aims at providing guidelines for managing type 2 Diabetes<br>• It mentions about the management of comorbid conditions namely hypertension,

					dyslipidaemia, obesity and TB
17	Guidelines for Prevention and Management of Stroke (Ministry of Health and Family Welfare, 2019)	2019	Ministry of Health and Family Welfare	Risk factors<br>Prevention and management of stroke<br>Integrated care	• The guide provides information about risk factors of stroke, its symptoms and pre/post stroke measures, stroke prevention and management<br>• It mentions on identifying risk factor for stroke such as hypertension, Diabetes, dyslipidemia, obesity, physical inactivity, unhealthy diet, tobacco and alcohol, other cardiac diseases such as rheumatic valve disease, atrial fibrillation, sickle cell diseases etc.
18	Ayushman Bharat - Comprehensive Primary Health Care through Health and Wellness Centres (Ministry of health and Family welfare)	2018	Ministry of Health and Family Welfare	Comprehensive care- preventive, promotive, curative, rehabilitative and palliative care<br>Comprehensive care	• It is an attempt to deliver comprehensive range of services spanning preventive, promotive, curative, rehabilitative and palliative care.<br>• HWC are envisaged to deliver expanded range services that go beyond maternal and child health care services to include care for non -communicable diseases, palliative and rehabilitative care, Oral, Eye and ENT care, mental health and first level care for emergencies and trauma, including free essential drugs and diagnostic services.
19	CPHC NCD Solution PHC Medical Officer User Manual (Ministry of Health and Family Welfare)	Not mention ed	Ministry of Health and Family Welfare	Screening and management of 5 common NCDs<br>- hypertension, diabetes, oral, breast and cervical cancers.	• This medical officer user manual details the workflows, work plans, protocols and dashboards for the GOI NCD technology solution regarding hypertension, diabetes, oral, breast and cervical cancers.
20	CPHC NCD Solution - NCD Application ANM User Manual	Not mention ed	Ministry of Health and Family Welfare	Screening and electronic recording of 5 common NCDs<br>- hypertension, diabetes, oral, breast and cervical cancers.	• It is a manual guiding the ANMs in using the application to create every individual's electronic health record who are screened.<br>• It also includes their workflows, work plans, protocols and dashboards for the GOI NCD technology solution regarding hypertension, diabetes, oral, breast and cervical cancers.
21	Module for ASHA on	Not	Ministry of Health and	Risk factors	• This module covers common non-

	Non-communicable Diseases (Ministry of Health and Family Welfare)	mention ed	Family Welfare	Prevention and health promotion	communicable diseases such as Hypertension, Diabetes and three common Cancers (Cervical, Breast and Oral Cancer) and associated risk factors.<br>• The focus of this module is on building the knowledge and skills of the ASHA in prevention and health promotion.
22	Module for Multi-Purpose Workers (MPW) - Female/Male on Prevention, Screening and Control of Common Non-Communicable Diseases	Not mention ed	Ministry of Health and Family Welfare	Health system level<br>Screening of risk factors<br>NCD management	• It includes screening and referral process of NCD(HT, DM, CVD, stroke, cancer) and<br>• risk factors associated with each condition. Management of NCDs with messages for lifestyle modification and medication at provider level.<br>• Health promotion to be undertaken at workplaces, in schools, families and at community level on diet, physical activity, cessation of alcohol and tobacco.<br>• Ensure participation of women in screening and treatment programmes owing to their vulnerable status in family and society.<br>• Ensure participation of elderly owing to higher risk of developing NCDs
23	Training Module for Staff Nurses on Population Based Screening of Common Non-Communicable Diseases	Not mention ed	NHM, Ministry of Health and Family Welfare	Training guidelines for staff nurses for population-level screening specifically diabetes, hypertension and certain cancers such as cervical cancer screening<br>Health system level	• Service delivery framework for screening NCDs (diabetes, hypertension) cancer(cervical, breast, oral)]<br>• Treatment and management of NCDs as a single disease condition.<br>• Patient education and diet counselling Modifiable and non-modifiable risk factors for NCDs
24	Training Module for Medical Officers for Prevention, Control and Population Level Screening of NCDs	2017	Directorate General of Health Services, Ministry of Health & Family welfare Government of India	Health system level<br>To prevent and control the risk of NCDs through population-based screening (PBS)where the medical officer has a vital role in inter-sectoral collaboration	• Role of medicals officers PBS of common NCDs with support from ANM and ASHAs.<br>• Includes Preparing training plan for supporting staff, IEC, Management and referral, monitoring and evaluation.<br>• Mobilize the community for screening,

					undertake health promotion, treatment adherence follow-up and create patient support groups
25	Non communicable disease Control Programme (AmruthamAarogyam)	2011-12	Directorate of Health Services, Government of Kerala	Reduction of NCD risk factors by providing health education, conducting screening (above 18 years)<br>-Health system level	• Treatment protocol for managing NCDs by providing medicines<br>• SWAAS clinics for control and prevention of OPD,<br>incorporated diabetes control and monitoring along with IHCI, Nayanamritham for diabetic retinopathy screening, COPD clinics, Diabetic Foot clinics, Stroke Management (SIRAS were some of the special programmes initiated<br>• Care of elderly for chronic NCDs during Covid pandemic
26	Makkalai Thedi Maruthuvam	2021	Directorate of Health Services, Government of Tamil Nadu	-Community-based and Institution-based interventions to improve compliance and control of the disease.<br>-Health system level	• The scheme ensures delivery of home-based health care services through a set of field functionaries.<br>• Services include screening and follow-up of NCD, home delivery of drugs (hypertension, diabetes), palliative, physiotherapy and COPD services.<br>• The existing NCD services provided at public health facilities in the State are brought under the umbrella of MTM for strengthening the referral and follow-up services<br>• Patients eligible for Home-based MTM services are referred to respective PHC.

Table S2 Summary of policy directions in the included document

***The levels of interventions marked in this table are the available policy actions or strategies available in the policy and not specifically with respect to NCD-multimorbidity.**

Sl. No	Document title	Country	Levels of intervention			
			Patient	Provider	HS	Macro
1	National Programme for Prevention & Control of Cancer, Diabetes, Cardiovascular Diseases and Stroke (Ministry of Health and Family Welfare, 2010)	India		√	√	√
2	Operational guidelines-National programme for health care of the elderly (Ministry of Health and Family Welfare, 2011)	India	√			
3	Twelfth Five-Year Plan (2012–2017) (Planning Commission, 2012)	India			√	
4	National Action Plan and Monitoring Framework for Prevention and control of NCDs. (Ministry of health and Family Welfare, 2012)	India		√	√	
5	Standard treatment guidelines- Hypertension Screening, Diagnosis, Assessment, and Management of Primary Hypertension in Adults in India (Ministry of Health and Family Welfare,2016a)	India		√	√	
6	Operational Framework Management of Common Cancers (Ministry of Health and Family Welfare, 2016b)	India		√		
7	Operational Guidelines on Prevention, Screening and Control of Common NCDs (Ministry of Health and Family Welfare, 2016c)	India		√		
8	Training Manual for Medical Officers on Reducing Risk Factors of NCDs (Ministry of Health and Family Welfare, 2016d)	India		√		
9	Training Manual for Community Health Workers on Reducing Risk Factors of NCDs (Ministry of Health and Family Welfare, 2016e)	India		√		
10	Training Manual for NCD Programme Managers at State and District Level	India		√		
11	National Health Policy (Ministry of Health and Family Welfare, 2017a)	India		√	√	
12	National multi-sectoral action plan for prevention and control of common non-communicable disease (Ministry of Health and Family Welfare, 2017b)	India		√	√	√
13	Hypertension Screening, Diagnosis, Assessment, and Management of Primary Hypertension in Adults in India (Ministry of Health and Family Welfare, 2017c)	India		√		
14	National Framework for Joint TB-Diabetes Collaborative Activities (Ministry of Health and Family Welfare, 2017d)	India		√		
15	Handbook for Counsellors - Reducing Risk Factors for NCDs (Ministry of Health and Family Welfare, 2017e)	India		√		√
16	ICMR Guidelines for management of type 2	India		√		

	diabetes 2018 (ICMR, 2018)					
17	Guidelines for Prevention and Management of Stroke (Ministry of Health and Family Welfare, 2019)	India		√		
18	Ayushman Bharat -Comprehensive Primary Health Care through Health and Wellness Centres (Ministry of health and Family welfare)	India		√		
19	CPHC NCD Solution PHC Medical Officer User Manual (Ministry of Health and Family Welfare)	India		√		
20	CPHC NCD Solution - NCD Application ANM User Manual	India		√		
21	Module for ASHA on Non-communicable Diseases (Ministry of Health and Family Welfare)	India		√		
22	Module for Multi-Purpose Workers (MPW) - Female/Male on Prevention, Screening and Control of Common Non-Communicable Diseases (Ministry of Health and Family Welfare)	India		√		
23	Training Module for Staff Nurses on Population Based Screening of Common Non-Communicable Diseases (Ministry of Health and Family Welfare)	India		√		
24	Training Module for Medical Officers for Prevention, Control and Population Level Screening of NCDs	India		√		
25	Non-Communicable Disease Control Programme (AMRUTHAM AROGYAM)-Kerala, India	India	√		√	
26	Makkalai Thedi Maruthuvam (Reaching out Health services to people)	India	√		√	

Sl. No	Document title	Country	Levels of intervention			
			Patient	Provider	HS	Macro
1	White paper for the transformation of the health system in South Africa (National Department of Health Republic of South Africa,2009)	South Africa	√		√	
2	Quality Improvement – the key to providing improved quality of care (National Department of Health Republic of South Africa,2012)	South Africa		√	√	
3	National mental health policy framework and strategic plan (National Department of Health Republic of South Africa,2013a)	South Africa	√		√	√
4	Strategic plan for the prevention and control of non-communicable diseases 2013-17(National Department of Health Republic of South Africa,2013b)	South Africa			√	√
5	National health insurance for South Africa (National Department of Health Republic of South Africa,2015a)	South Africa			√	
6	Implementation Guideline of Health Workforce Normative Guides and Standards for Fixed Primary Health Care Facilities (National Department of Health Republic of South Africa,2015b)	South Africa			√	
7	Strategy for the prevention and control of obesity in South Africa (National Department of Health Republic of South Africa,2015c)	South Africa	√		√	√
8	Taxation of sugar-sweetened beverages (National	South	√		√	√

	Department of Health Republic of South Africa,2015d)	Africa				
9	National Cancer Strategic Framework (NCSF) (National Department of Health Republic of South Africa,2017a)	South Africa	√		√	√
10	Policy Framework and Strategy for Ward-Based Primary Healthcare Outreach Teams (National Department of Health Republic of South Africa,2018a)	South Africa			√	√
11	Strengthening the South African health system towards an integrated and unified health system (National Department of Health Republic of South Africa,2018b)	South Africa	√	√	√	√
12	National Digital Health Strategy for South Africa (National Department of Health Republic of South Africa,2019a)	South Africa	√	√	√	
13	2030 Human Resources for Health Strategy: Investing in the Health Workforce for Universal Health Coverage (National Department of Health Republic of South Africa,2020a)	South Africa		√	√	√
14	Referral Policy for South African Health Services and Referral Implementation Guidelines (National Department of Health Republic of South Africa,2020b)	South Africa		√	√	
15	National strategic plan for the prevention and control of non-communicable diseases 2020-2025(National Department of Health Republic of South Africa,2020c)	South Africa	√	√	√	
16	ICSM model health Service Re-organisation (National Department of Health Republic of South Africa)	South Africa	√	√	√	
17	National user guide on the prevention and treatment of hypertension in adults at the PHC level (National Department of Health Republic of South Africa,2021)	South Africa		√		√

Sl. No	Document title	Country	Levels of intervention			
			Patient	Provider	HS	Macro
1	District health system management; To take care of the population: Elderly group, Chronic disease group and People with disabilities* (Health Promotion Foundation, 2013)	Thailand	√			
2	Guides to “Flat Belly” Organisation	Thailand			√	√
3	Prevention guidelines control diabetes and high blood pressure* (Department of Disease control, 2017a)	Thailand	√	√	√	
4	5-Year National NCDs Prevention and Control Plan (2017–2021) (Department of Disease control, 2017b)	Thailand	√		√	√
5	Proposal Prevention and Control of Non-communicable Diseases RTG-WHO Country Co-Operation Strategy January 2017–December 2021 (Department of Disease control, 2017c)	Thailand	√	√	√	√
6	(Draft) Thailand Healthy Lifestyle Strategic Plan	Thailand	√	√	√	√

	Phase II 5-Year Non-Communicable Diseases Prevention and Control Plan (2017-2021) and related Action Plan (Department of Disease control, 2017d)					
7	Service model for prevention and control of diabetes, high blood pressure (Department of Disease control, 2017e)	Thailand	√	√		
8	National Strategic Plan on Promotion of Physical Activity (2018–2030) (Ministry of Public health, 2018)	Thailand			√	√
9	Practice manual for tobacco addiction treatment in patients' chronic disease in Thailand*	Thailand		√		
10	Guidelines for health system development (Service Plan)<br>Integrated primary care system with people at the centre(Integrated, People-centred Primary Care)*(Ministry of Public health, 2020)	Thailand	√	√	√	√
11	Operational Guidelines NCD Clinic Plus & Online* (Department of Disease control, 2021)	Thailand	√	√	√	
	*Google translated from Thai to English					

Search strategy- Box S1

PubMed

(("multiple long-term conditions"[All Fields] OR "multi-morbidity"[All Fields] OR "multiple diseases"[All Fields] OR "multiple morbidity"[All Fields] OR "multiple chronic diseases"[All Fields] OR "multiple chronic conditions"[All Fields] OR "multiple illnesses"[All Fields] OR "multiple diagnoses"[All Fields] OR "multiple condition"[All Fields] OR "multiple long-term conditions"[All Fields] OR "multipathology"[All Fields] OR "chronic condition"[All Fields] OR "chronic diseases"[All Fields] OR "non-communicable disease"[All Fields]) AND 1000/01/01:2022/12/01[Date - Publication] AND (("policy"[All Fields] OR "programmes"[All Fields] OR "strategy"[All Fields] OR "reports"[All Fields] OR "action plans"[All Fields] OR "health plans"[All Fields]) AND 1000/01/01:2022/12/01[Date - Publication]) AND (("India"[All Fields] OR "South Africa"[All Fields] OR "Thailand"[All Fields]) AND 1000/01/01:2022/12/01[Date - Publication])) AND (1000/1/1:2022/12/1[pdat])

(("primary care"[All Fields] OR "re-organising"[All Fields] OR "strengthening primary care"[All Fields] OR "universal health coverage"[All Fields]) AND 1000/01/01:2022/12/01[Date - Publication] AND (("policy"[All Fields] OR "programmes"[All Fields] OR "strategy"[All Fields] OR "reports"[All Fields] OR "action plans"[All Fields] OR "health plans"[All Fields]) AND 1000/01/01:2022/12/01[Date - Publication]) AND (("India"[All Fields] OR "South Africa"[All Fields] OR "Thailand"[All Fields]) AND 1000/01/01:2022/12/01[Date - Publication]) AND 1000/01/01:2022/12/01[Date - Publication] AND (("multiple long-term conditions"[All Fields] OR "multi-morbidity"[All Fields] OR "multiple diseases"[All Fields] OR "multiple morbidity"[All Fields] OR "multiple chronic diseases"[All Fields] OR "multiple chronic conditions"[All Fields] OR "multiple illnesses"[All Fields] OR "multiple diagnoses"[All Fields] OR "multiple condition"[All Fields] OR "multiple long-term conditions"[All Fields] OR "multipathology"[All Fields] OR "chronic condition"[All Fields] OR "chronic diseases"[All Fields] OR "non-communicable disease"[All Fields]) AND 1000/01/01:2022/12/01[Date - Publication])) AND (1000/1/1:2022/12/1[pdat])

Box S2 India

Google (Advanced search)

Search term	Limits	Screened	
non-communicable disease programmes	filetype:pdf region: India	First 100	
multiple long-term conditions	file region: India type:pdf	27	
“multimorbidity policy”	file region: India type:pdf	0	
multimorbidity policy	file region: India type:pdf	First 100	
"Chronic disease programme"	file region: India type:pdf	2	
"chronic disease strategy"	file region: India type:pdf	0	
" multiple chronic disease strategy"	file region: India type:pdf	0	
"non-communicable disease health plans"	file region: India type:pdf	0	
non-communicable disease health plans	file region: India type:pdf	First 100	
“multi-morbidity OR policy" OR "multiple OR morbidity OR policy" OR "multiple OR chronic OR diseases OR policy OR " OR "multiple OR chronic OR conditions OR policy" OR "multiple OR illnesses OR policy" OR "chronic OR condition OR policy" OR "chronic OR diseases OR policy’" "multiple long-term conditions policy"	file region: India type:pdf	0	
multi-morbidity OR policy OR multiple OR morbidity OR policy OR multiple OR chronic OR diseases OR policy OR OR multiple OR chronic OR conditions OR policy OR multiple OR illnesses	file region: India type:pdf	First 100	

OR policy OR chronic OR condition OR policy OR chronic OR diseases OR policy multiple long-term conditions policy			
"Primary care programmes"	file region: India type:pdf	First 100	

Box S3

Digital copies of Indian public health policy documents were obtained on digital databases:

https://main.mohfw.gov.in/

https://main.mohfw.gov.in/?q=Major-Programmes/non-communicable-diseases-injury-trauma/Non-Communicable-Disease-II/National-Programme-for-Prevention-and-Control-of-Cancer-Diabetes-Cardiovascular-diseases-and-Stroke-NPCDCS

Digital copies of South African public health policy documents were obtained on digital databases: https://www.gov.za/documents/white-papers; https://www.health.gov.za/policies-and-guidelines/; https://libguides.lib.uct.ac.za/c.php?g=194637&p=5351707

Additional consultation was done with provincial stakeholders to ensure that relevant documents were included.
Digital copies of Thai public health policy documents were obtained by contacting team from Thailand.

Flow-diagram

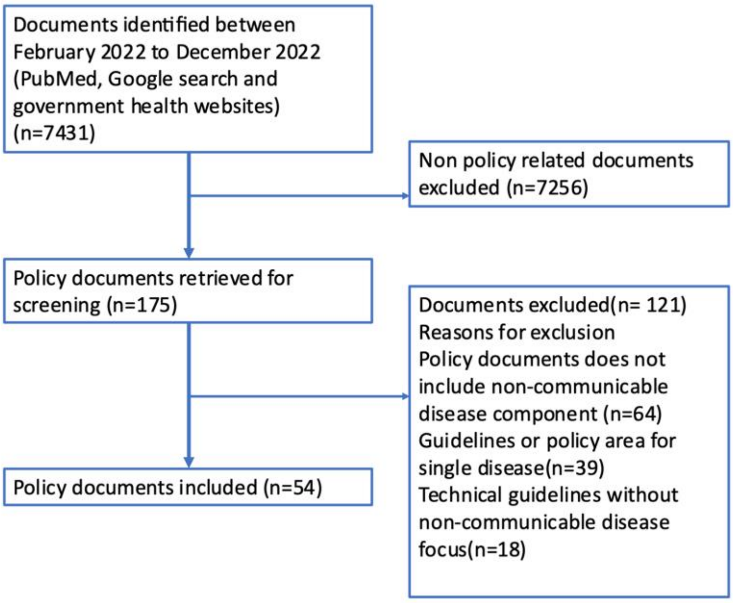

Fig S1: Document review flow-diagram
